# Making sense of the politics in the climate change loss & damage debate

**DOI:** 10.1016/j.gloenvcha.2020.102133

**Published:** 2020-09

**Authors:** E. Calliari, O. Serdeczny, L. Vanhala

**Affiliations:** a University College London, Department of Political Science & School of Public Policy, UK; b Climate Analytics, Berlin, Germany; c Euro-Mediterranean Centre on Climate Change and Ca’ Foscari, University of Venice, Italy

**Keywords:** Loss and damage, Negotiations, UNFCCC, Politics, International relations, Political ethnography, Multi-methods

## Abstract

The Warsaw International Mechanism for Loss and Damage (L&D) associated with Climate Change Impacts (WIM) was established in 2013 to advance i) knowledge generation; ii) coordination and iii) support to address losses and damages under the UNFCCC. So far, the work undertaken by the WIM Executive Committee (ExCom) has focused on enhancing understanding and awareness of the issue and promoting collaboration with relevant stakeholders. Delivering on the WIM’s third function on action and support has lagged behind, and ‘the political’ nature of L&D has often been blamed for this. Key terrains of contention among Parties have included the positioning of L&D governance vis-à-vis the adaptation space and struggles around state liability and compensation. As a way to facilitate discussion on implementation options, recent research has suggested de-politicising aspects of the L&D debate; yet we have very little insight into how the politics are understood within the realm of international L&D governance. This paper brings an analysis of ‘the political’ into the picture by identifying the complex and underlying issues that fuel contention within UNFCCC L&D negotiations. It gives centre stage to the way different framings of norms and material interests affect the debate, and challenges the tendency in current L&D literature to overlook the socio-historical and political underpinnings of this area of policy-making. We employ a qualitative multi-methods research design which draws on content analysis of 138 official Parties’ submissions and statements, 14 elite interviews with key current and former L&D negotiators and is built on a foundation of 3 years of participant observation at COPs and WIM meetings. We approach this data with a political ethnographic sensibility that seeks to explore how meanings are constructed within and across different sources of data. Our empirical results show that, rather than being a monolithic dispute, L&D catalyses different yet intertwined unresolved discussions. We identify five areas of contention, including continued disputes around compensation; conflicts on the legitimacy of L&D as a third pillar of climate action; tensions between the technical and political dimension of the debate; debates over accountability for losses and damages incurred; and the connection of L&D with other unresolved issues under the Convention.

## Highlights

• Deploying a political ethnographic sensibility can help to unpack the politics around L&D.
• L&D is not a monolith, but catalyses unresolved clashes under the UNFCCC.
• Compensation is a visible one but means different things to different Parties.
• Some Parties still question L&D as a third pillar of climate action.
• Unpacking the politics can enhance transparency in deliberations.

## 1 Introduction

In the global governance of climate change, discussion of climate-related loss and damage (L&D) emerged during the original development and drafting of the United Nations Framework Convention on Climate Change (UNFCCC). A proposal by the Alliance of Small Islands States (AOSIS) for an insurance pool to compensate vulnerable small island and low-lying developing countries for the impacts of sea level rise raised the spectre of climate change impacts that could not be adapted to (INC, 1991). Over the last decade the conceptualisation of L&D in the policy realm has been expanded to comprise the adverse effects associated with other slow onset and extreme events, and to focus on both monetizable impacts and intangible losses and damages which are increasingly referred to as ‘non-economic losses’ (NELs) (Tschakert et al., 2019). Research and policy work on NELs includes concerns with loss of biodiversity, territory, cultural heritage and encompasses the emerging issue of climate-induced human mobility. While there is no official definition of L&D under the UNFCCC, current scholarly understandings of L&D often emphasise the unavoidability and irreversibility of certain climate change impacts and the role played by constraints and limits to adaptation as drivers of adverse outcomes (McNamara and Jackson, 2019).

Vulnerable developing countries have historically argued that L&D constitutes policies, activities and finance that are ‘beyond adaptation’ and campaigned for compensation as a key remedy (Roberts and Huq, 2015). Developed nations have contested this legalistic framing and strongly rejected any notion of liability. Instead, they have sought to keep L&D firmly within the adaptation realm and emphasized overlaps with the disaster risk reduction (DRR) and humanitarian spheres (Calliari, 2016a, Vanhala and Hestbaek, 2016). A political compromise was eventually reached in the accompanying decision to the Paris Agreement (PA), by stating that the agreement’s Article 8 on L&D does not provide a basis for any liability or compensation. At the same time, the PA sanctioned the permanence in the post-2020 climate regime of the Warsaw International Mechanism for L&D associated with climate change impacts (WIM) which had been established in 2013 to advance: i) knowledge generation, ii) coordination and iii) support to address L&D (UNFCCC, 2014). The WIM is guided by an Executive Committee (Excom) whose activities are informed by a five-year rolling workplan that was carefully drafted by the members of the Excom. The workplan includes work on five strategic workstreams (WS) focusing on: A) slow onset events; B) NELs; C) comprehensive risk management approaches; D) human mobility; E) action and support, including finance, technology and capacity-building. To date the Excom’s work has focused on enhancing understanding and awareness of L&D and promoting collaboration with relevant stakeholders. Delivering on the WIM’s third function on action and support has lagged behind when compared with the other areas of the workplan (see, for instance, submission by Bhutan (2019)). Indeed, while work on WSs A-D has already kicked-off, activities under WS E were only considered in a more in-depth manner at the first meeting of the Excom in 2020 (Excom, 2019).

‘Politics’ has often been blamed for the slow pace of progress on L&D in the negotiations and in the implementation of related policies and decisions at the international level. The social scientific literature on L&D has been helpful in identifying some of the key lines of contention in processes of governance in this area. These include debates among Parties about the positioning of L&D policy-making vis-à-vis the adaptation space (‘L&D as within or ‘beyond’ adaptation’) and struggles around questions of justice and responsibility, including calls for liability and compensation (Calliari, 2016a, Vanhala and Hestbaek, 2016, Boyd et al., 2017). Some of these points have been addressed and partly resolved through the adoption of the PA. For many commentators the elaboration of a separate article on L&D has provided recognition of the issue in international law, which was seen as a significant outcome for developing country Parties (Sharma, 2017). On the other hand, the explicit exclusion of liability and compensation claims under the PA (a relatively rare type of provision in international environmental agreements) was seen as an important victory for Global North states. Yet before the ink on the agreement was dry, international law scholars warned that ‘all options remain[ed] open’ under the climate regime for small island developing States (SIDS) and other vulnerable countries to satisfy the concerns underlying their calls for compensation and liability (Mace and Verheyen, 2016). All of this suggests that the current approach to L&D in the climate change governance architecture remains ambiguous (Calliari, 2016a, Vanhala and Hestbaek, 2016) and concerns about the financial implications of addressing L&D persist, given the slow nature of progress on this topic and the focus on market-based and private sector instruments within WIM discussions (WIM Excom, 2016). As a result, fostering action and support for L&D remains challenging: it is hard to agree on what the scope of L&D programmes should be, and how and by whom they should be financed (Gewirtzman et al., 2018).

As a way to facilitate progress, recent research and policy briefs have suggested that aspects of the L&D debate could be depoliticised. For instance, Boyd et al. (2017) – drawing on interview data with a wide range of stakeholders involved in the L&D discussion – find that, while there are significant disagreements on L&D, there is no simple polarization between those seeking compensation and those wishing to avoid paying for it. Similarly, Byrnes and Surminski (2019) explicitly call for depoliticising the L&D discussions in the submission of the LSE Grantham Institute to the WIM review in 2019. Mechler et al. (2019) also identify a number of ways of ‘grounding’ the ‘so far highly political debates’ associated with L&D. However, most of the analyses that suggest de-politicisation of L&D do not go further in unpacking the politics. Recent research has suggested that there is a tendency in current L&D literature to overlook the socio-historical and political determinants of the debate (McNamara and Jackson, 2018). This – combined with a more general lack of research on L&D policy development in political science and in international relations – constitutes a barrier to developing a deeper understanding of the challenges in this area of global climate governance.

In this paper we contribute to remedying this gap by asking the following: how is ‘the political’ nature of L&D framed by those immersed in this stream of governance at the international level? By showing how those most closely involved in the process frame the politics, we offer a more nuanced picture of the debate. We provide a novel understanding of L&D policy-making at the international level by engaging with those at the very heart of contentious action: negotiators. The paper focuses on the micro-politics of L&D but at the macro level: the sites chosen for data generation include both COP negotiations and WIM Excom meetings. This choice rests on the fact that multilateral negotiations remain the primary site where L&D is discussed and the Excom meetings are a key venue for the implementation of L&D-related decisions. While both state and non-state actors are already carrying out L&D activities at the national level and on the ground, what is (not) decided at the international level is likely to have implications for the scale and nature of practical measures to be taken. As such, we trace the way these international ‘insiders’ articulate relevant concepts and we explore which factors they identify as fuelling contention within the L&D debate, as well as the contested interpretations of their implications. We do this through a multi-method qualitative approach rooted in an ethnographic sensibility (Bayard De Volo and Schatz, 2004, Fujii, 2010, Schatz, 2009). In particular, we rely on a three-pronged data collection strategy consisting of a qualitative content analysis of 138 official submissions to the UNFCCC from 2008 to 2016; 14 elite interviews with key current and former L&D negotiators representing a wide range of perspectives and country groupings and building on a foundation of 3 years of participant observation of COP and WIM Excom meetings from 2016 to 2019 which helped to identify the issues we focus upon in this article.

To be clear, our aim is not to identify points of contention to simply suggest they be de-politicised but rather to problematize some existing assumptions among practitioners and scholars and to enhance transparency, thus contributing to the delineation of a common symbolic framework where competing perspectives can be constructively and democratically managed (Mouffe, 2005). Specifically, recent ethnographic research on international organisations and the role of the international bureaucracy has highlighted a trend towards ‘post-political forms of regulation and government’ (Mouffe, 2005) and ‘anti-politics’ (Ferguson, 1990). For example, Merry (2006) and Riles (2000) both found that while transparency is now an explicit value of the international community, the ‘performance of “transparency”’ can often ‘hide as much as it reveals’ through idiosyncratic speaking practices, heavy usage of acronyms and intense haggling around terminology resulting in heavily coded and opaque ‘technical’ texts. States are now expected to strategically advance their geopolitical interests within the UN in a seemingly non-politicised way (Müller, 2013). By applying a political ethnographic sensibility, we seek to shed some light on how this applies to the UNFCCC context.

We do not treat ‘politics’ as a dirty word and we suggest that there is a need to openly and rigorously analyse the distributional – and thus political – consequences of any deliberative process (including L&D negotiations). By shifting attention to these dynamics in studying the L&D framework we thus complement observations made by Javeline, 2014, Eriksen et al., 2015 that adaptation – far from being a neutral, technical and managerial process – is based on contestation of what counts as ‘adaptive’ for different groups and implies differentiated outcomes in terms of vulnerability and adaptive capacity. We suggest these considerations are equally – if not more – applicable in the L&D realm. We also suggest that the lack of political science and international relations research on L&D policy-making represents a barrier to effectively understanding, developing and implementing L&D policies because all aspects of L&D are fundamentally political. Even seemingly highly technocratic solutions to losses and damages to, for example, infrastructure, agriculture, bio-diversity and to more intangible losses, like loss of culture or loss of livelihoods, all depend on political agreements being reached and political institutions implementing those agreements across different jurisdictions and levels of governance. By bringing the politics back into the picture, this article gives centre stage to the way in which norms (such as ideas of justice, fairness and responsibility) and material interests are framed by negotiators and how these framings shape the debate and the possibilities for progress.

## 2 Methods and material

Interpretive approaches that take processes of meaning making seriously are increasingly deployed in the study of global environmental politics. On one hand, anthropologists and sociologists have ventured to explore international organisations and have shown how seemingly mundane and ordinary interactions, as well as those heavy with symbolic meaning and expressions of power, can reveal something important about the culture of international organisations and the way individuals think and operate within them (Abélès, 2011, Müller, 2013). Yet, there has been relatively little research on the UNFCCC using these techniques and analytical lenses (Barnes et al., 2013). On the other hand, political science and international relations scholars have tracked the emergence of ideas around particular topics, explored the role of science in international policy-making and examined how norms become embedded in international discourses (Campbell et al., 2014, Suiseeya, 2014, Suiseeya and Zanotti, 2019, Witter et al., 2015). They have also contributed to methodological innovation in the field through the development of collaborative event ethnography (Brosius and Campbell, 2010, Corson et al., 2013). Longitudinal ethnographic approaches have been also employed to explore justice claims-making in climate negotiations (Thew et al., 2020). Yet the issue of L&D governance has not yet been scrutinised in this way but may be particularly well suited to it given the political challenges in this area.

We draw on an interpretive approach to better understand different framings of ‘the political’ in the area of L&D. Through rendering explicit these meaning-making processes, we are able to identify the issues at the heart of contention. We adopt a political ethnographic sensibility in the way that we approach our interlocutors and our data in our quest to ‘*glean the meanings that the people under study attribute to their social and political reality*’ (Schatz, 2009). While the question of what is ‘political’ about ‘political ethnography’ has tended to be addressed differently across disciplinary divides, there is a relative consensus that most definitions include the struggle to define its jurisdiction, which also characterises our approach here (Auyero, 2006, Baiocchi and Connor, 2008, Benzecry and Baiocchi, 2017, Hagene, 2018, Pachirat, 2018, Schatz, 2009). With this political ethnographic sensibility we generated and analysed different types of data. We employed content analysis of official Party submissions and elite interview as data generation strategies, and built on a foundation of participant observation that guided the inquiry presented here. Across disciplines there is growing challenge to the hegemony of participant observation as ethnography, particularly among scholars who are involved in ‘studying up’ i.e. studying the culture, behaviour, and world views of elites (Nader, 1969). As Stepputat and Larsen (2015) note, accessing and analysing texts and interviews can address some of the challenges of accessing fieldwork sites, informants and knowledge, and constitutes what Gusterson calls ‘polymorphous engagement’(Gusterson, 2008).

As a first step, we accessed and analysed Parties’ submissions to the UNFCCC from 2008 to 2016. Submissions and statements prior to 2008 are not electronically available, yet we chose this year as an appropriate starting point considering that L&D was mentioned for the first time in a COP decision in 2007. We collected relevant submissions and statements to technical bodies (AWG-LCA, SBI) as well as opening and closing statements to intersessional climate change conferences (the so-called ‘SBs’). We collected a total of 426 documents, of which 298 are submissions to the AWG-LCA (2008–2012), 43 are submission to the SBI (2011–2013) and 85 are opening and closing statements to the SBs (2014–2016). From this initial pool, we identified 138 referring to L&D by searching for the following keywords (in English, Spanish and French): *loss, damage, loss and damage, compensation, liability, historical responsibility, debt, remedy, justice.* We manually checked that these keywords were employed in discussions related to L&D and not, for instance, REDD, mitigation (e.g. compensation for mitigation costs), or response measures. Our participant observation allowed us to enter the field with some concepts that we prioritised and that have been central in L&D debates, but the approach we took to understanding these concepts was open and guided by our data.

Using an iterative research design and abductive reasoning that moves continuously between theory and data, we searched for evidence of the two controversies in L&D negotiations that have been identified in existing literature and in participant observations: i) L&D scope (within or beyond adaptation) and ii) historical responsibility/liability and compensation. Using Nvivo, we coded statements within the two categories to identify Parties’ positions on the controversies and to trace their evolution over time. The resulting mapping was used to construct a historical account of the development of these controversies and to complement and contrast with the data we generated through elite interviews.

As a second step, we conducted 14 in-depth semi-structured interviews between July 2017 and March 2019 with key negotiators and former negotiators on L&D under the UNFCCC. Respondents were identified among those meeting at least one of the following criteria: i) being a current or former member of the WIM Executive Committee (ExCom); ii) having participated at COPs where L&D milestones were reached (Cancun, Doha, Warsaw and/or Paris). An effort was made to ensure geographical representativeness and to capture the positions of the main negotiating groups. We focused exclusively on negotiators because of their unique, situated angles on the issue at hand. Most studies of L&D policy making at the UNFCCC level have involved a much wider array of actors (observers, experts and secretariat staff) in their data-gathering approaches, which is useful for the types of questions those studies addressed related to the full range of conceptualisations and definitions applied in the L&D field (Boyd et al., 2017, Vanhala and Hestbaek, 2016). In this study, with its focus on how negotiators themselves make sense of the politics in this realm, data from those types of sources would render the picture we are trying to paint opaque. It is also worth noting that gaining access to negotiators from across Party groupings is a significant achievement (one which was facilitated in part by our positionality – discussed further below).

Interviewees included 4 negotiators from developed countries, i.e. former Annex-1 countries in the UNFCCC lingo (Interviewees 1, 2, 3 and 4), and 10 from developing countries, i.e. former non-Annex 1 countries: 3 from Africa (Interviewees 5, 6 and 13); 1 from Latin America (Interviewee 7); 1 from the Arab Group (Interviewee 8); 4 from both Caribbean and Pacific Ocean Small Island Developing States (Interviewees 9, 10, 11, 12) and 1 representative of the G77 and China (Interviewee 14). Participants included negotiators of varying degrees of involvement in the L&D process, ranging from 3 to more than 10 years of experience. Interviews were conducted over the phone, skype and in person, and lasted between 30 min and 1.5 h. After gaining consent, all interviews were recorded and transcribed by two research assistants.

Continuing in the iterative vein of this research, negotiators were asked in semi-structured interviews to i) identify 3 topics in L&D negotiations likely to generate tensions; and ii) critically reflect on three thematic areas where progress on L&D could be enhanced, i.e. Slow Onset Events (SOEs), Permanent Losses (PL), and Finance. We identified these areas by scrutinizing current technical work under the WIM and through participant observation at WIM ExCom and COP meetings. In particular, SOEs were chosen as a contrast in light of the predominant focus on comprehensive risk management and responses to extreme weather events within the WIM ExCom’s work. The inclusion of permanent losses was motivated by the discrepancy between their mention as a bullet point in Article 8 and the lack of a related working area within the 5-year workplan of the mechanism. Finally, finance was selected because it has long been an area of contention.

Interviewing negotiators poses specific challenges in terms of ‘trustworthiness’ (Pachirat, 2018). As a way to mitigate the risk of interviews becoming ‘negotiations by proxy’, we asked those negotiators that seemed to be justifying or elaborating on a negotiating position to critique his or her own case, so as to explore the extent to which research participants were able to move away from their own perspectives. The mapping of official Parties and groupings’ positions in Step 1 underpinned this interview strategy. By reconstructing the evolution of official Parties’ statements over time, we were able to recognise – and hence query – those statements merely mirroring official positions. It is worth noting that there will always be room to further query whether the data reflects ‘sincere’ or ‘strategic’ perspectives. This is true of all ethnographic work to some extent but we do not deem this limitation of critical significance for our findings given the fact that we are interested in how these actors frame and make sense of particular issues *as negotiators*.

A third prong of our approach involved our participant observation at the COPs and WIM Excom meetings from 2016 to 2019, which served as a foundation upon which to build our other data generation work. This observation activity provided us with an in-depth understanding of which areas have proven most difficult to reach agreement on as well as those that are overlooked in terms of the five-year workplan and the report of the Excom to the COP each year. This knowledge allowed us to develop more effective, targeted interview scripts and to effectively code the Party submissions.

Finally, two key tenets of an interpretivist approach are worth addressing here, which entail seeing research as a reflexive practice and the importance of acknowledging our positionality. This concerns the ways in which the researchers’ identities (and interlocutors’ perceptions of those identities) are not independent from the research process and findings. In this case, the three authors share some traits but are also differently situated. We are all women from the global North who at the time of research had already published peer-reviewed research on the topic of L&D. While two of us have been active in this field primarily as researchers, including as observers at COPs and ExCom meetings, the third has been (and continues to be) actively involved in the negotiations as an advisor to SIDS and Least Developed Countries (LDC) negotiators. It is likely that this position and associated relationships both facilitated access to the negotiators we sought to interview and may also have influenced the responses they gave in interviews. We made all of our interlocutors aware of this information beforehand, and it is possible that this may have shaped responses, particularly for those from the Global North. Playing this kind of dual role (as expert and/or participant and researcher) is not uncommon among anthropologists of international organisations who regularly have to navigate the tensions between insider and outsider roles (see e.g., Bendix, 2013).

## 3 Results

This section presents the results of the two prongs of structured data generation and analysis which are also informed by insights from our ethnographic observations. The first part presents the mapping of the official Party submissions, the second presents the results of the analysis of the interview data.

### 3.1 Mapping of Parties’ positions in official submissions

At a high-level, the mapping of Parties’ positions affirms what has been found in previous research: that debates about the relationship between L&D and adaptation governance – whether L&D is something separate and additional to adaptation or part and parcel of it – and contestation over understandings of historical responsibility, state liability and compensation have dominated this area of negotiations (Boyd et al., 2017, Calliari, 2016a, Vanhala and Hestbaek, 2016). However, this analysis for the first time allows us to gain a deeper understanding of how these debates have evolved over time and how and why some Party positions have changed at particular points in time. One point to bear in mind with the analysis is that this picture emerges from official submissions and hence should be understood as a reflection of strategic documents rather than sincere positions (though this does not preclude that the two may be aligned).

#### 3.1.1 The relationship between L&D and adaptation governance

The first major issue concerns the relationship between L&D and adaptation governance. In the run up to the Copenhagen COP in 2009, developing countries predominantly framed L&D as an adaptation issue. For instance, AOSIS (2009) – in Article 3 of its proposal for a Copenhagen protocol - included L&D in the list of ‘*adaptation actions*’. The same point was made by Algeria on behalf of the African Group, 2009, Brazil, 2009, India, 2009, Nicaragua on Behalf of Guatemala, Dominican Republic, Honduras, 2009, and Tuvalu (2009). The Multi-Window Mechanism to Address Loss and Damage proposed by AOSIS in 2008 was also presented among the ‘*means to incentivize adaptation actions on the basis of sustainable development*’ (AOSIS, 2008).

With the Cancun Adaptation Framework established in 2010, and an institutional anchorage thus given to the issue of adaptation, a conceptual distinction between L&D and adaptation began to emerge in the context of the work programme on L&D (2011–2012). Developing countries began to consistently refer to L&D as impacts ‘*beyond adaptation*’ (Bolivia (Plurinational State of), Ecuador, China, El Salvador, Guatemala, Thailand, 2012, Gambia, 2012, Gambia, 2011, Ghana, 2012), whereas in their submissions developed countries would clearly situate it within adaptation and disaster risk management approaches (Canada, 2011, EU, 2012, Norway, 2011). For instance, the USA (2011) emphasised how the work programme should support ‘*approaches such as risk reduction, micro-insurance, and macro-insurance*’.

What is striking is that in WIM ExCom meetings there is rarely discussion of this issue: members tend to be quiescent on the relationship between L&D and adaptation. There are several potential reasons for why this is the case. It may be that this issue is one that is perceived to be dealt with in the negotiations rather than within the Excom which is focused more on policy development rather than political agreement, or it may be that the existence of the WIM ExCom itself is understood to be a symbolic or institutional acknowledgment of L&D as a separate sphere of activity. However, this quiescence at the ExCom meetings contrasts with the continuing relevance of the question in the negotiations. For example, at COP 25 in Madrid in 2019 the issue was consistently raised by developing country Parties in both informal consultations on the WIM and in the plenary sessions particularly in relation to the need for finance for L&D activities as ‘separate and additional to’ finance for adaptation (Fieldnotes, COP 25 Madrid, December 2019).

#### 3.1.2 Notions of state liability and compensation

The second major issue identified through the mapping of Party submissions concerns notions of state liability and particularly mentions of compensation as a remedy for historic responsibility. While calls for compensation are now firmly situated within the L&D space this has not always been the case. Most developing countries started to advance compensation claims in the context of adaptation. Bolivia (2008) called for developed countries to ‘*pay compensation for the past, present and future damage caused by the impacts of climate change*’. This position was further elaborated in pointing to an ‘*adaptation debt”* owed by developed countries to developing countries (Bolivia, 2009, Venezuela, 2010). On the same note, Bangladesh (2009) proposed to include the ‘*setup of a rapid financing window (…) including compensation mechanism*’ in the suite of ‘*adaptation activities*’ in the post-2012 regime. Similar language was employed by Guatemala on Behalf Of Belize, Costa Rica, Dominican Republic, El Salvador, Guatemala, Honduras, 2009, Micronesia, 2009, Guyana, 2009 referring to, for example, a ‘*compensation mechanisms for adaptation*’.

AOSIS was the first grouping to explicitly link compensation and L&D. In a submission to the ‘Dialogue on long-term cooperative action to address climate change by enhancing implementation of the convention’, it called for considering ‘*how vulnerable countries will be compensated for loss and damage associated with climate change impacts that is not avoided by adaptation funding under the Convention*’ (AOSIS, 2007). This point was expanded in the Multi-Window Mechanism a year later (AOSIS, 2008), which suggested a holistic approach to the needs of vulnerable developing countries by bringing together ‘*tools to address adaptation, financial risk management and risk transfer, and loss and damage*’. The mechanism consisted of: i) an insurance component to manage financial risk from extremes; ii) a rehabilitation/compensatory component to address negative impacts from SOE; iii) a risk management component to facilitate and inform i) and ii). Arguably off the back of this proposal, compensation as an issue began to be tied explicitly to L&D by other developing countries (Algeria on behalf of the African Group, 2009, Bolivia, 2010, Brazil, 2009, Colombia, 2009, Islands, 2009, Grenada on behalf of AOSIS, 2010, Guatemala on Behalf Of Belize, Costa Rica, Dominican Republic, El Salvador, Guatemala, Honduras, 2009, India, 2009). AOSIS reiterated the three-fold approach of the Multi-Window Mechanism in discussions under the SBI (AOSIS, 2012). In that context, Bolivia, Ecuador, China, El Salvador, Guatemala, Thailand, Philippines and Nicaragua also made reference to compensation for the adverse impact of SOEs – in the form of a ‘solidarity fund’ – and rehabilitation (Bolivia (Plurinational State of), Ecuador, China, El Salvador, Guatemala, Thailand, 2012).

Interestingly, calls for compensation were largely abandoned after the establishment of the WIM in 2013. Among the few calls for compensation that continued to crop up was the Central American Integration System’s (SICA, in Spanish) demands for the establishment of a financial component of the WIM and the provision of rehabilitation and compensation for L&D (SICA, 2014). Reference to compensation was also made by AOSIS and the G77 + China in the proposal for a ‘Climate Change Displacement Coordination Facility’. Among the performed functions, the facility was to provide ‘*compensation measures for people displaced by climate change*’ – a provision that was dropped without excessive clamour on the road to Paris (Calliari, 2016a). Most of the calls for compensation we identified in our analysis of the submissions were concentrated before 2013, when discussing the establishment of an institutional mechanism to address L&D under the Convention (what eventually became the WIM). Based on participant observation at ExCom meetings from 2016 to 2019 we also note that the term ‘compensation’ has not been used in the meetings that have been open to observers which we attended. At COP 21, the decision text includes an explicit exclusion of liability claims and compensation requests which is understood to be a part of a trade-off by developing countries for a dedicated L&D article in the PA. However, mentions of compensation reappeared in a number of interpretative declarations to the instruments of ratification of the PA (UNTC, 2016). Bolivia, the Philippines, Nauru, Marshall Islands, Cook Islands, Solomon Islands and Tuvalu emphasised how the application of the PA shall in no way constitute a renunciation of any rights under international law concerning state responsibility for the adverse effects of climate change, and that no provision can derogate from principles of general international law or any claims or rights concerning compensation due to the impacts of climate change.

### 3.2 Deconstructing the politics in the L&D debate

While the analysis above shows the evolution of the two key political debates in L&D negotiations which have also been the focus of existing scholarly analysis, our interviews highlight other issues which negotiators attribute meaning and importance to as well. Certainly, the liability and compensation issue is still understood to be the most contentious issue by negotiators from across the developed-developing country divide (8 out of 14 research participants raised it unprompted during interviews; 4 developed; 4 developing). However, an important original finding that we present here are the differing understandings of the compensation issue and interpretations of how they interact with other elements of climate governance both within the L&D debate and beyond. The relationship between adaptation and L&D governance was also brought up in an unprompted way by a number of the research participants. The issue of who is responsible for addressing losses and damages – which is a debate that has been identified in previous research (Calliari, 2016a, Vanhala and Hestbaek, 2016) – was also raised by negotiators as was consideration of how ‘the technical’ nature of L&D interacts with ‘the political’. A surprising finding was the persistence of questions about the legitimacy of L&D as a separate stream of work within the UNFCCC even years after the establishment of the WIM. Finally, a factor that tends to be overlooked in most research on L&D is its relationship with other tracks of the climate change negotiations.

#### 3.2.1 The ghost of compensation and its multivalence

Compensation and liability were the themes most often mentioned by negotiators when asked to identify the top three issues likely to generate tensions in L&D talks. As one small island states negotiator puts it, “*anything that hints of liability and responsibility creates tensions*” (Interviewee 10). While the language in the decision accompanying the PA pushed compensation claims out of the UNFCCC’s remit, both developed and developing countries’ Interviewees recognise the possibility for the remedy to be pursued through other legal channels (Interviewees 2 and 10), a point already stressed by several small island states in their interpretative declaration to the PA (UNTC, 2016). Yet, the formal exclusion of compensation and liability claims from the UNFCCC process does not seem to imply that the ghost of compensation will stop fidgeting in negotiating rooms (Interviewee 7). In the view of an African negotiator, the clause ‘*doesn’t seem to be giving them* [i.e. developed countries] *the comfort that they want. It makes the discussion really difficult*’, because “*at the back of their minds, when we don’t even mean it they are just reading it into [what we say]* (Interviewee 13).

The interviews interestingly highlight the existence of different understandings or framings of ‘compensation’ suggesting the multivalence of the concept. A Caribbean negotiator distinguishes multiple historical interpretations of compensation among developing countries: in the research participant's view low lying islands facing ‘*an immediate existential threat*’ considered compensation as the only prompt remedy, while larger developing countries showed longer term concerns (e.g. desertification) and sought a ‘*support mechanism (…) to help them address those concerns over time*’ (Interviewee 11). In an African negotiator’s words, compensation broadly refers to support to ‘*help the vulnerable countries to develop capacity’* through ‘*capacity building*’ and ‘*technology transfer*’, and not simply ‘*asking for money’* (Interviewee 6)*.* Another African colleague argues that even when referring to ‘rehabilitation’, as reflected in the Cancun decision, in developed countries’ negotiators’ minds that may sound like ‘compensation’. Yet, as the Interviewee states, ‘*rehabilitation could mean several things*’ and not (just) asking ‘*developed countries to come and pay’* (Interviewee 5).

#### 3.2.2 Legitimacy of L&D as a separate issue

In line with the official country submissions, most developed country negotiators explicitly talk about L&D as belonging to the realm of adaptation (Interviewees 1, 2, 3). On the part of developing countries, the separation between L&D and adaptation is instead assumed – at least among our interlocutors (e.g. Interviewee 11) - and taken as the starting point for negotiations. As the analysis of official submissions shows, struggles around definitions characterised the work programme on L&D (2011–2012), but seem to be currently set aside. A developing country respondent notes that: ‘*there is no longer the need to debate the very premise of L&D: not because people have come to full agreement, but because there are ways to by-pass those issues* (Interviewee 14). Yet, interviews reveal a more profound and substantial set of questions being asked: are climate-related losses and damages recognized as a problem by all Parties sitting at the negotiation table? Is talking about these losses and damages perceived as legitimate? As a developed country negotiator puts it, there is ‘*a problem that nobody agrees on … what the nature of the problem really is, and people are trying to twist (…) the definition so that it actually fits the interests they want to achieve’* (Interviewee 1). Other developed countries’ negotiators (Interviewees 2, 3 and 4) implicitly or explicitly share this point of view. For those who explicitly share this view this seems to be largely rooted in an attempt to be ‘*practical (…) and see what works on the ground*’ (Interviewee 3), which in turn reveals difficulties in conceptually distinguishing L&D governance from DRR, adaptation and humanitarian work (Interviewee 2). This reasoning goes as far as not ‘*understand[ing] in what way loss and damage would be different or separate from mitigation or from adaptation*’ and in considering the consensus reached in various COP decisions and in Article 8 of the PA as purely politically motivated (Interviewee 1). The analysis of the interviews raises questions about whether developed countries really understand what developing countries want (Interviewee 4) – or instead make assumptions about this. This results in developing countries feeling their requests for action and support in addressing L&D are being “*push[ed] back*” (Interviewee 10). This is an issue that has also dominated some of the Excom meetings that we have observed and was a particular feature of informal consultations and plenary sessions at COP 24 and 25.

#### 3.2.3 Whose responsibility?

Interviews reveal tension around how the interplay between international cooperation and the agency of national governments should be articulated. In other words: how much of a role should national governments and the international community each play in addressing the adverse impacts of climate change? How should the development choices of national governments, which might affect the exposure and vulnerability of their population, be factored into decisions about the balance of responsibility? The point about “*accountability*” (Interviewee 3) and “*responsibility*” (Interviewee 4) of countries in exacerbating the impact of disasters is a leitmotif in developed countries’ framing. A developed country negotiator exemplifies the point by recalling how years ago the Marshall Islands hosted the Pacific Island Forum (PIF) by ‘*building right along the coastline*’ and how decisions about ‘*building along really vulnerable areas*’ ‘*still keep being made*’ (Interviewee 2). Even more bluntly, another developed Party negotiator highlights that ‘*we are starting from a world where people are put at risk, and where governments have made a lot of decisions not to protect their own people*’ (Interviewee 3). To a certain extent this position is recognized by their developing country counterparts, with the latter emphasising nevertheless the “*added constraint*” imposed by climate change which is “*making what was a bad situation worse”* (Interviewee 11).

#### 3.2.4 Two sides of the same coin? L&D as a technical or political problem

Nearly all negotiators (12 out of 14; 8 developing; 4 developed) refer directly or indirectly to two levels of discussion within negotiations: a *technical level* at which pragmatic solutions may be sought, and a *political level* that would ideally be bracketed to allow for informed debate and partial progress on the ground. Direct references are made either when the existence of two levels is framed as a problem or challenge (e.g. ‘*those two levels have totally different logics, and totally different dynamics*’, Interviewee 1) or when the need to have separate technical and political discussions is flagged (e.g. ‘*Number two [solution] would be to work on the aspects of loss and damage from a technical perspective*’, Interviewee 8). Indirect references are made when either one of the levels is singled out explicitly (e.g. ‘*from a technical perspective I would totally agree*’, Interviewee 11) or the discussions are described as political (e.g. ‘*it is still a very political issue’*, Interviewee 13).

The (alleged) multi-level nature of the L&D debate is acknowledged and problematized by negotiators. A small island negotiator traces the lack of progress on permanent losses to the fact that the “*WIM is still at a political level*” and lacks “*real experts*” (Interviewee 9). A developed country negotiator reports of having been shocked when joining L&D negotiations in realizing that, together with colleagues, they “*were never working at a technical level*” (Interviewee 4). There is a general consensus, supported by existing research (Thomas and Benjamin, 2018), that knowledge and data gaps are a significant obstacle towards action. As a Small Island State negotiator reports: “[Permanent losses] *is a complex issue and I guess it is also one of the explanations for why we have not looked into it. Not, that we are ignoring them but understand the issues around it before we get into it”* (Interviewee 12).

At the same time, there seems to be agreement that the technical and political dimensions cannot be divorced and need to be addressed concurrently (Interviewees 2 and 11). As a developed country negotiator underscores, addressing only one side will not be effective and technical responses alone will ‘*not address the concern of the other side which is a much more political high-level one*’ (Interviewee 1). Advances on L&D might thus be obtained by reconciling what is “*technically and politically feasible*” (Interviewee 2).

#### 3.2.5 L&D as symptom of other problems

With different degrees of explicitness, respondents connect controversies in the L&D discussion to wider existing disputes within the UNFCCC and beyond. The most evident, recognized by both developed and developing countries, is the lack of ambition in mitigation, adaptation and support. The link with mitigation is cited most commonly by developed countries (“*if the progress on mitigation is considered sufficient then I suppose the loss and damage issue will not be such a problem anymore”,* Interviewee 1). Yet, some developing countries also recognise the role of major emerging economies as emitters, and the way this is causing discomfort to small island states in the G77 + China group (Interviewee 8). The complexity of South-South dynamics seems to have affected G77 + China position on the topic. As highlighted by a developing country negotiator, ‘*there was a common understanding that compensation was not part of the G77 position*’ (Interviewee 14). From a different angle, the point is also made by a developed country negotiator that refers to the opposition by China as a ‘*major emitter*’ to any ‘*liability framework’* (Interviewee 2).

Issues of support are raised by developing country research participants. In particular, finance is felt to have always been “*a problem in the whole Convention*” (Interviewee 7), with developed countries also acknowledging this point (Interviewees 2 and 3). Yet, controversies over support seem to epitomize a more generally felt weakness in the way North-South assistance is deployed. While developed countries suggest increased cooperation with the DRR, humanitarian and development communities as a complementary way to systematize and gather funding, the proposal provokes a general disappointment with humanitarian aid which is especially felt by small islands states. There is an impression of ‘*money tend[ing] to go back to those very same countries’* where the support came from and that it ‘*does not build technical capacity within [recipient] countries’* which makes it then ‘*difficult to quantify and qualify the support that [has been](…) actually received on the ground* (Interviewee 11)*.*

## 4 Discussion

There is now a scholarly consensus that the liability-compensation dispute is important in explaining the slow pace of progress in L&D negotiations and implementation of related activities. Our analysis builds on these findings by showing that despite the exclusion of compensation-related discussions in the post-PA era, these calls have a long legacy and shape understandings of what is being asked for in terms of action and support. It also interestingly reveals that compensation is not a monolithic concept and research participants tended to refer to it in different ways – not necessarily with a legalistic or liability framing in mind. This is important for two interrelated reasons. First, for some there is a static understanding of what ‘compensation’ means whereas for others its explicit exclusion from the UNFCCC’s mandate has changed the meaning of this term. This reveals the way assumptions or simplifications of other Parties’ objectives can shape and ultimately hinder the debate. It also shows the different ways in which the use of this term resonates with different audiences when it is used for either strategic reasons or out of ‘good faith’. Second, it shows potential to go beyond stated mutually-exclusive positions (for or against compensation) and recognise the different meanings negotiators attribute to the concept and the assumptions they may make about the underlying interests or concerns of others. Principled negotiation theory has shown the importance of focusing on the core concerns implied in official positions, which often refer to basic human needs like security, material well-being, recognition, sense of belonging, and control over one’s own destiny (Fisher et al., 1991). For instance, calls for compensation by Pacific SIDS can be understood not just a request for finance but rather an expression of the existential threat they face and the desire for recognition of its unjust root causes. This example suggests that focusing on core concerns offers the potential of moving away from the current configuration of L&D negotiations as a win-lose negotiating game to be solved through compromise or domination. Future research investigating core interests and concerns underlying official positions could enhance understanding of how, and to what extent, they could be reconciled or how common goals could be devised.

Controversies around liability and compensation also impact on discussions around the relationship between L&D and adaptation. As our historical account of Parties’ positions shows, struggles around a conceptual distinction explicitly emerged in the context of the L&D Work Programme. Developing countries began to call for separate provisions under the UNFCCC and increasingly identified compensation as a key component of L&D-specific responses, while developed countries denied the need for provisions other than adaptation and rejected any notion of responsibility. As a way forward, Parties ‘agreed to disagree’ on a strict definition of L&D within the UNFCCC. Recent research has shown how such constructive ambiguity was helpful in institutionalizing L&D under the UNFCCC (Calliari, 2016a, Serdeczny, 2017, Vanhala and Hestbaek, 2016). It allowed for depolarizing the debate by letting every Party have its own meaning reflected in Decision 2/19 establishing the WIM, and later in Article 8 of the PA. Yet, it also had the side-effect of preventing constructive discussion around the framing of the problems to be addressed and the resulting space within which to develop solutions. Our results also highlight that contention can go as far as questioning the very existence of the concept of L&D and its legitimacy within the architecture of the UNFCCC. This begs the question of whether agreement on a shared definition of L&D would be helpful. Some have argued that shared meaning is necessary to advance progress (Page and Heyward, 2017), while other have suggested that any attempts to devise a definition would inspire antagonistic sentiments and lead to political deadlock (Boyd et al., 2017). By interviewing negotiators, we did not find evidence of any preferred option in this respect. Future research could extract useful insights and lessons-learnt from the way adaptation moved from an undefined and politically sensitive issue within negotiations (Schipper, 2006) to a pillar of climate action on par with mitigation in the context of the Paris Agreement.

Our analysis also importantly points to the need for scholarship in this area to consider L&D governance in conjunction with other issues within the UNFCCC. These include the equitable distribution of responsibility for climate action (mitigation, adaptation and support) as it should be embedded in the Common but Differentiated Responsibilities and Respective Capabilities (CBDR-RC) and equity principles. The implementation of these principles – extensively discussed in terms of mitigation (Rajamani, 2016) and, at least to some extent in regards to adaptation funding (Winkler and Rajamani, 2014) – poses unique challenges for L&D-related action. When considering the relationship between mitigation activity and the L&D debate – i.e. losses and damages as a result of insufficient ambition in mitigation – whether CBDR-RC should be interpreted and operationalised in light of evolving national circumstances or based on a static understanding of these remains unresolved. The G77 + China includes emerging economies, which officially support L&D as a group position, but are regarded as big emitters by its most vulnerable members. Our results show, for instance, how the group compromised to keep compensation claims out of G77 + China positions, with this indicating discomfort by some Parties’ in supporting the position. Similar dynamics emerge when dealing with CBDR-RC in the adaptation realm, where the principle is operationalised by recognizing the vulnerability of particular groups, communities and ecosystems; by allowing for flexibility for countries in their adaptation actions; and by calling for financial support for developing countries (Rajamani, 2018). In that context, the polarization of interests between emerging economies and the most vulnerable states is epitomised by struggles over the reference to ‘particularly’ vulnerable developing countries when defining potential beneficiaries of L&D support, as exemplified by the dynamics within the G77 + China group at COP22 (Calliari, 2016b). These disputes within the G77 + China group importantly point to more intricate political struggles around L&D, rather than a simplistic juxtaposition between developed and developing countries.

Yet, our results show that the issue of responsibility might acquire another (unexpected) connotation in the L&D debate. The main conundrum to overcome here is how to strike a balance between the need of the international community to support adaptation efforts and the accountability of national governments to protect their citizens by not increasing their exposure or vulnerability. Developed country respondents consistently referred to the role of national institutions in exacerbating the impact of disasters in developing countries. However, it is very difficult to understand how this complex and context-specific interplay between ‘responsibilities’ across governance scales – the national and international – could be accounted for and operationalised in the climate change regime. While the focus on countries’ primary responsibility for protecting their own people and infrastructure is strong in a blueprint like the Sendai Framework for Disaster Risk Reduction (UN, 2015), it seems difficult to reconcile with treaty provisions on historical responsibility for anthropogenic climate change.

This paper also highlights the added value of adopting a political ethnographic sensibility. By combining analysis of texts, participant observation techniques and interviews with negotiators, we are able to uncover the polyvalent meanings of important concepts such as ‘compensation’ and ‘adaptation’ that are core to the L&D debate. Understanding the ways in which these concepts are deployed politically and the multiplicity of meanings that negotiators attribute to these (and other) ideas would not be possible through interviews or participant observation alone. Therefore in order to truly begin to understand ‘the political’ in this area of climate governance it is useful to both be immersed within the political context where this dialogue happens and explore how the insiders themselves approach key issues.

Finally on a related, theoretical level we argue in contrast to existing scholarship that de-politicising L&D may not be feasible or even desirable. First, de-politicisation is *per se* a highly political process (Bettini, 2013), as it implies covering up or repressing the conflictual aspects that are at the essence of the definition and deliberation of an issue. As such, removing ‘the political’ has the very political effect of reaffirming existing dominant relations. Within the UNFCCC, this would mean re-producing current power asymmetries in climate negotiations (Schroeder et al., 2012). Second, even assuming a de-politicised deliberation process, the resulting actions will have distributional - and thus political - effects. The growth of delegated bodies and the growing prevalence of rationalistic-technocratic discourse in the realm of L&D may be an avenue for progress but they will also have political impacts that should be considered by social scientists. Finally, de-politicisation may not be desirable in that it may hinder possibilities for discussion, debate and potential resolution. Recognising (rather than repressing) political conflicts can help to create a common ground for constructive debate. In short, through acknowledgment of the very political nature of the issue ‘antagonists may be turned into agonists’ (Mouffe, 2005). Recognising the political nature of L&D has thus the very practical merit of making normative and material stumbling blocks in the debate explicit, which then may allow for the possibility of meaningful deliberation and action.

## 5 Conclusions

L&D is arguably one of the most contentious issues to have emerged within climate negotiations in recent years. While the lion’s share of scholarly and media attention has been paid to the very visible disputes around compensation and liability, our paper offers a more fine-grained understanding of the politics involved. An important, original finding we present are the differing understanding of the compensation issue, and how these interact with other elements of climate governance both within and beyond the L&D debate. By showing the multiple and contested meanings of the idea of ‘compensation’ and how this shapes Parties’ objectives, we highlight opportunities to go beyond stated mutually-exclusive positions to single out less confrontational issues from irreducibly antagonistic ones. Another key and surprising finding the paper brings to research on L&D concerns the persistence of questions about the legitimacy of L&D as a third pillar of climate policy, even years after the establishment of the WIM and Article 8 of the PA. The paper further unpacks tensions between the technical and political dimension of the debate; disputes over accountability for losses and damages incurred; as well as the relationship of L&D with other tracks of climate negotiations – all factors that tends to be overlooked in most research on L&D. It also importantly helps to refine our understanding of the political struggles around L&D by moving away from a simplified representation of the debate as a Global North *vs* Global South issue.

This refined understanding of the politics around L&D would have not been possible without adopting an interpretivist perspective. A key feature of the paper is that it provides a new understanding of L&D dynamics by engaging with those at the very heart of negotiations, i.e. negotiators. From a methodological point of view, this stresses the need to both be immersed within the political context where these dialogues unfold and to explore how insiders approach key issues if we want to understand the political challenges in this area of climate governance. Finally, from a related theoretical perspective, this research highlights the opportunities associated with not treating ‘politics’ as a dirty word and to openly and rigorously analyse the normative and distributional – and thus fundamentally political – implications of deliberative processes within the UNFCCC.

## Declaration of Competing Interest

The authors declare that they have no known competing financial interests or personal relationships that could have appeared to influence the work reported in this paper.
